# Case report: Hemorrhagic fever with renal syndrome presenting as hemophagocytic lymphohistiocytosis

**DOI:** 10.3389/fmed.2022.1096900

**Published:** 2022-12-12

**Authors:** Maarten A. J. De Smet, Simon Bogaert, Alexander Schauwvlieghe, Amélie Dendooven, Pieter Depuydt, Patrick Druwé

**Affiliations:** ^1^Department of Intensive Care Medicine, Ghent University Hospital, Ghent, Belgium; ^2^Department of Hematology, Ghent University Hospital, Ghent, Belgium; ^3^Department of Pathology, Ghent University Hospital, Ghent, Belgium

**Keywords:** hemophagocytic lymphohistiocytosis, hemorrhagic fever with renal syndrome, hantavirus, critical care, communicable diseases *Puumala virus*-associated hemophagocytosis

## Abstract

Hemophagocytic lymphohistiocytosis may occur in patients with genetic predisposition and in sporadic cases due to malignancy or infection. We describe a 49-year old man with hemorrhagic fever, type 1 respiratory insufficiency and acute kidney injury. Diagnostic work up showed a hyperinflammatory syndrome, hypertriglyceridemia, hemophagocytosis, very high ferritin and significantly elevated sCD25. The findings were compatible with hemophagocytic lymphohistiocytosis based on the HLH-2004 criteria. Serological testing indentified *Puumala virus* as the causal pathogen. The patient was successfully treated with pulse corticosteroids, intravenous immunoglobins and supportive therapy.

## Introduction

Hemophagocytic lymphohistiocytosis (HLH) is a rare syndrome, characterized by an aberrant hyperinflammatory and hyperferritinemic immune response that is driven by T cells and excessive proliferation of activated histiocytes. HLH occurs as a primary genetic form or secondary to malignancy, infections, immunodeficiency and rheumatological or auto-immune disorders (1). Infection-associated HLH may be caused by viral, bacterial, parasitic or fungal pathogens. Viral infections, such as Epstein-Barr virus, cytomegalovirus, herpes simplex virus, varicella zoster virus, influenza, SARS-CoV-2, and HIV, are known to trigger HLH. Rarely, HLH is associated with hemorrhagic fever with renal syndrome (HFRS) caused by *Hantaan, Seoul, Dobrava/Belgrade, or Puumala virus*. Six case reports have been described with only one identifying *Puumala virus* as the causal pathogen (2–7). Here, we describe a case of HLH secondary to HFRS caused by *Puumala virus*.

## Case report

A 49-year old man presented to the emergency department following a week of night sweats and fever up to 39°C. He complained of right upper quadrant pain, dyspnea, headache, photophobia, and diplopia. The patient was a metal worker and kept guinea pigs, hamsters and rabbits. No other animal contact was reported. Two weeks before presentation, he received a second dose of the BNT162b2 mRNA COVID-19 vaccine. There was no history of recent or travel to endemic areas. He smoked actively, drank alcohol sporadically and used no drugs. There were no known allergies and the family history, including active or recent infections, was negative. On examination, heart rate was 83 beats per minute, blood pressure 118/81 mmHg, respiratory rate 18 per minute, oxygen saturation 98% without supplemental oxygen and temperature 38°C. Lung auscultation revealed bibasal inspiratory crepitations. The right upper quadrant was painful upon palpation without signs of hepatosplenomegaly or peritonitis. No other significant abnormalities were present. Hemoglobin was 11.1 g/dL, platelet count 27 × 10^3^/μL, white blood cell count 20.4 × 10^3^/μL with neutrophilia, serum creatinine 4.3 mg/dL, aspartate aminotransferase 87 U/L, alanine aminotransferase 59 U/L, gamma-glutamyl transferase 72 U/L, alkaline phosphatase 102 U/L, lactate dehydrogenase (LDH) 538 U/L, CRP 78 mg/L, ferritin 10631 μg/L, triglycerides 518 mg/dL, albumin 24 g/L, fibrinogen 360 mg/dL (Table 1). Computed tomography of head, chest and abdomen revealed ascites without other abnormalities. Blood and urine cultures were incubated. A SARS-CoV-2 PCR was negative.

**TABLE 1:** Results of laboratory testing.

	Admission	Day 3	Day 5	Day 10	Discharge	1 Month	3 Months
**Blood**							
ESR (mm/h) (<15)	2	15					
CRP (mg/L) (<5)	57	78.2	34.5	4	3.1	<1	<1
Ferritin (μg/L) (20–280)	10631	6999	4947	1453		634	313
D-dimer (ng/mL) (<500)	2420	4680					
Fibrinogen (mg/dL) (200–400)		360	287	199		190	197
Erythrocytes (10^6^/μL) (4.4–5.8)	3.45	3.8	3.45	3.74	3.47	3.62	3.67
Hb (g/dL) (13.5–17)	11.1	12.6	11.1	12.2	11.4	12	12.5
Hct (%) (39.9–51)	32	35.3	32	35.2	33.9	37.9	36.4
PLC (10^3^/μL) (143–325)	27	76	129	381	324	332	408
Citrate PLC (10^3^/μL) (143–325)	20	70					
WBC (10^3^/μL) (4.3–9.6)	20.4	17.6	14.35	20.72	17.19	16.78	7.64
Neutrophils (10^3^/μL) (1.9–5.9)	15.2	11			12.59		
Lymphocytes (10^3^/μL) (1.2–3.4)	2.1	3.6					
Monocytes (10^3^/μL) (0.3–0.8)	1	2.2					
Eosinophils (10^3^/μL) (0.03–0.4)	0.4	0.3					
Basophils (10^3^/μL) (0.02–0.1)	0.1	0.1					
Creatinin (mg/dL) (0.7–1.2)	1.2	4.3	3.74	1.02	0.84	0.74	0.78
Total bilirubin (mg/dL) (0.2–1.3)	0.6	0.4	0.4	0.6	0.6	0.4	0.5
Total protein (g/L) (64–83)		47	64	65	58		
Albumin (g/L) (35–52)		24	25	29			
AST (U/L) (<37)	87	68	114	35	43	23	18
ALT (U/L) (7–40)	59	48	77	107	111	39	16
γGT (U/L) (<64)	72	51	203	174	171	75	19
AP (U/L (30–120)	102	90	114	110	110	76	68
LDH (U/L) (105–250)	500	538	467	302	286	269	193
NT-proBNP (pg/mL) (<125)		5466					
Triglycerides (mg/dL) (58–327)		518	287	395			
sCD25 (U/mL) (458–1997)		3602					
NK cells (/μL) (90–600)		129.5					
NK perforin (%)		86.9					
NK cell function (degranulation test)		Normal					
Leptospira antibodies		Negative					
Puumala IgG (<0.8)		3.1		3.5			
Puumala IgM (<0.8)		8.8		7.2			
Puumala PCR		Negative					
Panhantavirus PCR		Negative					
**Urine**							
Puumala PCR		Negative					
Panhantavirus PCR		Negative					

ESR, erythrocyte sedimentation rate; CRP, C-reactive protein; Hb, hemoglobin; Hct, hematocrit; PLC, platelet count; WBC, white blood cells count; AST, aspartate aminotransferase; ALT, alanine aminotransferase; γGT, gamma-glutamyl transferase; AP, alkaline phosphatase; LDH, lactate dehydrogenase; NT-proBNP, N-terminal prohormone of brain natriuretic peptide; sCD25, soluble CD25; NK, natural killer; PCR, polymerase chain reaction.

The patient was started on ceftriaxone and vancomycin because of suspected meningoencephalitis. After 2 days, persistently high inflammation prompted a switch of vancomycin to doxycycline. Blood and urine cultures were repeated and remained sterile.

The patient was transferred to the ICU because of type 1 respiratory failure due to pulmonary edema and bilateral pleural effusions requiring High Flow Nasal Oxygen (HFNO). In addition, he developed nephrotic syndrome as documented by arterial hypertension, microscopic hematuria and proteinuria (5.2 g protein/g creatinine). Because of suspected HLH, intravenous immunoglobins (IVIGs, 1 g/kg daily for 3 days) and pulse corticosteroids (dexamethasone 20 mg on the first day, followed by 10 mg daily) were started.

**FIGURE 1:**
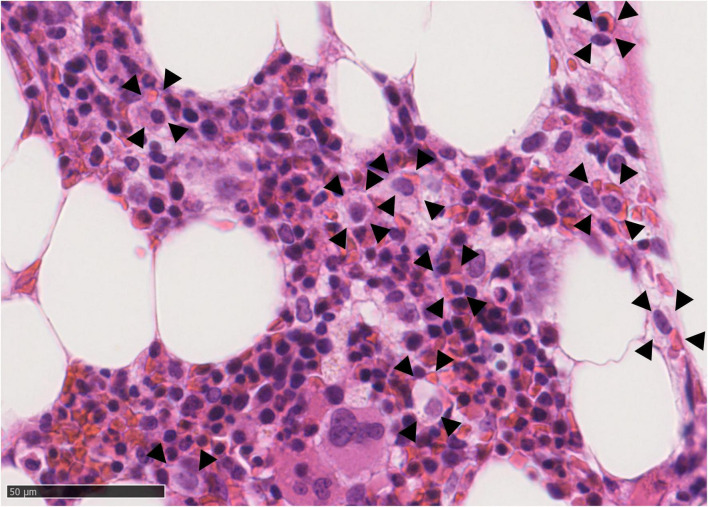
Bone marrow biopsy (Hematoxylin and Eosin stained, 400×) showing histiocytes (arrows) engulfing red blood elements compatible with hemophagocytosis.

Further workup showed NT-proBNP of 5466 pg/mL with normal cardiac structure and function on transthoracic echocardiography. A lumbar puncture was acellular and showed LDH of 47 U/L and total protein of 224 mg/dL without monoclonality. Cerebrospinal fluid culture and viral PCRs for *herpes simplex virus, varicella-zoster virus, enterovirus, Epstein-Barr virus and cytomegalovirus* remained negative. Serum and urinary protein electrophoresis did not show monoclonality. Bone marrow aspiration showed reactive changes without evidence of malignancy. Bone marrow biopsy revealed hemophagocytosis (Figure 1). Kidney biopsy showed slight acute tubular damage. Soluble CD25 (sCD25) was 3602 pg/mL. Natural Killer cell function was normal without perforin deficit.

Hemophagocytic lymphohistiocytosis was diagnosed because of a suggestive clinical phenotype in combination with a hyperinflammatory syndrome, fever, hypertriglyceridemia, hemophagocytosis, very high ferritin and significantly elevated sCD25 (1). *Puumala virus* serology was positive on hospital day 3 and 10 (Table 1), confirming HFRS. *Hantavirus* PCR was negative on blood and urine. Serology for other relevant infections and auto-immune disorders was negative (Supplementary Table 1). Exome sequencing could not reveal mutations at HLH foci or for immunodeficiency syndromes. Additional lab work did not uncover any immunocompromising factors.

Two days after the start of IVIGs and corticosteroids, serum creatinine improved and the patient could be weaned off HFNO therapy. Dexamethasone was tapered over 3 months. Currently, the patient is asymptomatic, with normal kidney function and without inflammation.

## Discussion

Hemophagocytic lymphohistiocytosis is a rare syndrome, characterized by an aberrant hyperinflammatory and hyperferritinemic immune response that is driven by T cells and excessive proliferation of activated histiocytes. HLH may occur as a primary genetic form or secondary to infections, malignancy, immunodeficiency and rheumatological or auto-immune disorders. While variations in HLH-associated genes may also play a role in adult-onset HLH, primary HLH occurs mostly in children. Secondary HLH is most common in adolescents and adults as a result of acquired immune dysfunction in response to infections, malignancies, and immune disorders (1).

Infection-associated HLH may be caused by viral, bacterial, fungal or parasitic pathogens. Viral infections, such as Epstein-Barr virus, cytomegalovirus, influenza, SARS-CoV-2, and HIV, are known to trigger HLH (8, 9). Rarely, HLH is associated with hemorrhagic fever with renal syndrome (HFRS) (2–7). HFRS is a zoonotic viral disease caused by Old World hantaviruses, occurring in Asia and Europe, such as *Hantaan, Seoul, Dobrava/Belgrade or Puumala virus*. In contrast, New World hantaviruses, occurring in the Americas, may cause hantavirus pulmonary syndrome (HPS). To our knowledge, no reports of HLH in HPS have been described in the literature. Transmission occurs through contact with saliva from bites or inhalation of aerosolized excrements of asymptomatically infected rodents. Clinical presentation varies from subclinical to fatal. After an incubation period of 2–4 weeks, patients may show non-specific symptoms such as fever, chills, headache, abdominal pain, nausea, and vomiting. After the initial period, hemorrhagic complications as well as renal dysfunction may occur that are associated with mortality. The diagnosis of hantavirus infection is generally confirmed by serological testing since viral RNA usually disappears from the circulation a few days after symptoms start. Six case reports have described HLH secondary to hantavirus infection with only one identifying *Puumala virus* as the causal pathogen (2–7). The bank vole is the reservoir for *Puumala*. However, here, clinical history suggests transmission through domesticated rodents. Experimental research shows hamsters may be subclinically infected with *Puumala*, but naturally occurring transmission has not been previously described (10). To the best of our knowledge, this is the first report describing such a possibility of transmission for *Puumala virus*.

Hemophagocytic lymphohistiocytosis is diagnosed based on the HLH-2004 criteria (Supplementary Box 1) (1). Firstly, heterozygosity for HLH-associated mutations or gene defects of other immune regulatory genes together with clinical findings associated with HLH may diagnose the syndrome. Alternatively, five of the following findings must be present for the diagnosis of HLH: fever ≥ 38.5°C, splenomegaly, peripheral blood cytopenia (at least two of the following: hemoglobin <9 g/dL, platelets < 100,000/μL, absolute neutrophil count < 1000/μL), hypertriglyceridemia ≥265 mg/dL and/or hypofibrinogenemia ≤ 150 mg/dL, evidence of hemophagocytosis, low or absent NK cell activity, ferritin ≥ 500 ng/mL and elevated sCD25 ≥ 2400 U/mL. In this case, HLH diagnosis could be confirmed based on five criteria: fever, hypertriglyceridemia, hemophagocytosis, very high ferritin and significantly elevated sCD25. Additionally, functional and genetic testing did not reveal pathogenic variants associated with HLH or primary cytotoxicity defects, suggesting secondary HLH.

Given the coagulation abnormalities in this case, dissiminated intravascular coagulation (DIC) has to be considered as a complication. Signs of DIC are common in hantavirus infection. In *Puumala virus* infections, 28% of patients may be diagnosed with DIC. DIC was also associated with more severe disease and may also complicate HLH (11).

The previously mentioned diagnostic criteria do not always need to be met in order to initiate treatment. Early suspicion is important to initiate HLH-specific therapy in critically ill or deteriorating patients. The HScore may be used to assess the probability of HLH (Supplementary Box 2) (1). This score incorporates points for immunosuppression, fever, hepatosplenomegaly, triglyceride level, ferritin, AST and fibrinogen, cytopenias and presence of hemophagocytosis on bone marrow aspiration. An HScore ≥ 250 confers a 99% probability of HLH, whereas a score of ≤90 confers a < 1% probability of HLH. In this case, HScore was 196 with a probability of having HLH of 85% based on fever, thrombycytopenia, very high ferritin, triglycerides >350 mg/dL, fibrinogen ≤ 250 mg/dL and AST ≥ 30 U/L. When hemophagocytosis was additionally shown to be present, HScore increased to 231 with an HLH probability of 98%.

Hemophagocytic lymphohistiocytosis-specific therapy may consist of pulse corticosteroids, IVIGs, etoposide, cyclosporin A and/or biologics such as the interleukin-1 antagonist anakinra or the interleukin-6 antagonist tocilizumab (1). In patients with central nervous system involvement, methotrexate may be added (1). The HLH-94 protocol suppresses activated T cells and inflammatory cytokine production. It is the mainstay treatment in children up to 18 years of age, where genetic causes of HLH are enriched. The HLH-94 protocol consists of corticosteroids, cyclosporin A and etoposide. Additional intrathecal therapy is suggested in case of progressive neurological symptoms or persistent abnormal cerebrospinal fluid after 2 weeks of therapy.

The heterogeneity of adult HLH prohibits a single uniform protocol. Accordingly, treatment in adults cannot be standardized and needs tailoring according to the underlying condition and HLH-initiating trigger (infection, malignancy, auto-immune/auto-inflammatory conditions, drug-induced, other causes) (1). The treatment experience of HFRS-induced HLH is very limited. Successful results have been reported with supportive therapy, IVIGs, corticosteroids and etoposide (2–7). Additionally, in infection-associated HLH, the causal pathogen should be identified rapidly and empiric or directed therapy should be initiated. In this case and supplementary to HLH-specific therapy, the patient was treated with broad-spectrum antibiotics, on a suspicion of sepsis. Antibiotic treatment was stopped when cultures remained negative.

## Conclusion

Hemophagocytic lymphohistiocytosis may be associated with HFRS caused by *Puumala virus* infection and may be successfully treated with supportive therapy, IVIGs and pulse corticosteroids.

## Data availability statement

The original contributions presented in this study are included in this article/Supplementary material, further inquiries can be directed to the corresponding author.

## Ethics statement

Ethical review and approval was not required for the study on human participants in accordance with the local legislation and institutional requirements. The patients/participants provided their written informed consent to participate in this study.

## Author contributions

All authors listed have made a substantial, direct, and intellectual contribution to the work, and approved it for publication.
