# Supplementary material for: Case report: Hemorrhagic fever with renal syndrome presenting as hemophagocytic lymphohistiocytosis
Source: Front Med (Lausanne). 2022 Dec 12;9:1096900. doi: 10.3389/fmed.2022.1096900 (PMC9790895; doi:10.3389/fmed.2022.1096900)
Supplement: Supplementary file 1 [file Data_Sheet_1.docx]

Supplementary Material

| **Test** | **Result** |
| --- | --- |
| **Serum** | |
| **Anti-CCP (U/mL) (<7)** | 0.4 |
| **Rheumatoid factor (U/mL) (0-40)** | <11.3 |
| **ANF** | Negative |
| **ANCA** | +1, atypical |
| **Anti-GBM (U/mL) (<7)** | <1.9 |
| **Parvovirus B19 IgG (<0.9)** | 45 |
| **Parvovirus B19 IgM (<0.9)** | 0.7 |
| **Leptospira antibodies** | Negative |
| **Bartonella henselae IgG (<1/320)** | <1/320 |
| **Bartonella henselae IgM (<1/320)** | <1/320) |
| **Brucella IgG** | Negative |
| **Brucella IgM** | Negative |
| **Coxiella Burneti IgG** | Negative |
| **Coxiella Burneti IgM** | Negative |
| **Toxoplasma IgG (U/mL) (<3)** | 0.1 |
| **Toxoplasma IgM (<1)** | 0.23 |
| **EBV IgG (<0.5)** | <0.5 |
| **EBV IgM (<0.75)** | 24.5 |
| **Mycoplasma pneumoniae IgG (<1)** | <1 |
| **Mycoplasma pneumoniae IgM (U/mL) (<10)** | <10 |
| **Rubella IgG (U/mL) (<10)** | 37.6 |
| **Rubella IgM (<1)** | 0.1 |
| **Measles IgG (U/mL) (<13.5)** | >300 |
| **Measles IgM (<0.9)** | 0.1 |
| **Mumps IgG (U/mL) (<9)** | <5 |
| **Mumps IgM (<1.1)** | 2.2 |
| **CMV IgG (U/mL) (<6)** | 5.1 |
| **CMV IgM (<1)** | 1.16 |
| **HIV antigen + antibodies** | Negative |
| **Hepatitis A IgG (U/L) (<1)** | 7.11 |
| **HBsAg** | Negative |
| **HBsAL (U/L) (<10)** | 0 |
| **HBcAL** | Negative |
| **HCV antibodies** | Negative |
| **EBV DNA, CMV DNA** | Not detected |

**Supplementary Table 1.** Results of other serological testing

| **The diagnosis of HLH can be established if either 1 or 2 are fulfilled.** | **Present in the index case** |
| --- | --- |
| 1. A molecular diagnosis consistent with HLH | No |
| 1. Five out of eight diagnostic criteria for HLH are fulfilled:  - Fever - Splenomegaly - Cytopenias (affecting ≥2 lineages in the peripheral blood): Hemoglobin <9 g/dL, platelets <100 000/µL, Neutrophils <1000/µL - Hypertriglyceridemia (≥265 mg/dL or ≥3 mmol/L) and/or hypofibrinogenemia (≤150 mg/dL) - Hemophagocytosis in bone marrow or spleen or lymph nodes - Low or absent NK cell activity - Ferritin ≥500 µg/L - sCD25 ≥2400 U/mL | Yes No No  Yes  Yes  No Yes Yes |

**Supplementary box 1.** HLH-2004 diagnostic criteria

| **Parameters** | **Criterion points** | **Points in the index case** |
| --- | --- | --- |
| Known underlying immunosuppression | 0 (no) or 18 (yes) | 0 |
| Temperature (°C) | 0 (<38.4), 33 (38.4–39.4), 49 (>39.4) | 33 |
| Organomegaly | 0 (no), 23 (hepatomegaly or splenomegaly), 38 (hepatomegaly and splenomegaly) | 0 |
| Cytopenias | 0 (1 lineage), 24 (2 lineages), 34 (3 lineages) | 0 |
| Ferriting (µg/L) | 0 (<2000), 35 (2000-6000), 50 (>6000) | 50 |
| Triglycerides (mg/dL or mmol/L) | 0 (<1.5), 44 (1.5-4), 64 (>4) | 64 |
| Fibrinogen (mg/dL) | 0 (>2.5) or 30 (≤2.5) | 30 |
| AST (U/L) | 0 (<30) or 19 (≥30) | 19 |
| Hemophagocytosis on bone marrow aspirate | 0 (no) or 35 (yes) | 35 |
| Total | 337 | 231 |

**Supplementary box 2.** HLH-probability calculator (HScore) (http://saintantoine.aphp.fr/score/)
